# The Critical Period of Homœopathy

**Published:** 1878-12

**Authors:** H. M. Paine

**Affiliations:** Albany


### Selection.

### THE CRITICAL PERIOD OF HOMŒOPATHY.

By H. M. Paine, M. D., Albany.

[The following article, taken from the *Homœopathic Times*, is here reproduced as an interesting evidence of the fact of the decadence of homœopathy. The tidal wave, which long since ebbed away from the shores of the old world, and which but recently was at its flood in America, is evidently doomed to complete disappearance. The misstatements here made relative to regular medicine, require no refutation; nor need the readers of this journal be told that the term "allopathic," which is so frequently repeated below, always has been and always will be utterly repudiated by the true physician. Ed.]

Dr. E. M. Hale published an article in the November (1877) number of the *American Homœopathist*, entitled, "A Critical Period for Homœopathy," in which he clearly sets forth the danger to our school from the adoption of homœopathy by old school physicians, *without an open acknowledgment to that effect.* This article is strongly supported by indisputable evidence, and clearly points out the influences which are accomplishing the destruction of the homœopathic school, *as a separate and influential body of medical men.*

Accessions to our ranks are derived from only *two* sources: those who are educated under homœopathic auspices, and converts from the so-called regular school. Of the first class named, those who have graduated from our own medical schools, there were the present year only three hundred and nineteen,* a number so small as to be scarcely sufficient to fill the places made vacant by death and other causes. It is plainly apparent, therefore, that recruits from this source must be largely increased, or else we

* *Investigator*, May 1, 1878.

must depend chiefly on the second class mentioned, viz., converts from the so-called regular school.

On even a cursory examination in this direction, the result is exceedingly unpromising. Those of us who were participants in the contest between the two principal rival schools can vividly recall the scenes which occurred twenty and even fifteen years ago. Then there were constant accessions to our ranks from those of our opponents. Desertions were so numerous as to impair the strength of allopathic legal organizations, and, in some localities, seriously threaten their existence. At the present day the exodus has nearly ceased. The comparatively few converts who are willing, openly, to admit their belief in homœopathy, may be numbered by tens, while formerly there were hundreds.

In looking for the causes which have brought about this result, we find that they are mainly due to the adoption of a wise and liberal policy on the part of the allopathic school toward its own members. During the past ten years instances of the resort to discipline for practicing homœopathically, have been of very rare occurrence. The so-called regular school has virtually abolished all rules having reference to the infliction of a penalty regarding matters of medical belief. Its members are now freely allowed the privilege of believing and practicing all systems of treatment extant. It has practically adopted the wise suggestions recommended by Dr. Dunham, in his admirable address, delivered before the American Institute of Homœopathy, in June, 1870, regarding "Liberty of opinion and action."

This prudent and liberal policy has enabled our opponents to retain very large numbers of physicians who would otherwise have united with homœopathic medical societies.

By this radical change of base our rivals have largely promoted the popularity and numerical strength of their own school. Strange as it may appear, is it not on account of the adoption of this liberal policy, that the accessions to the number of homœopathic physicians are mainly within the membership of the so-called regular school? While the number of physicians who are willing to publicly announce their belief in homœopathy may be diminishing, the number who are *actually* practicing homœopathically is *steadily increasing*.

I am acquainted with a number of allopathic physicians residing in this city, all of them in good standing in their own medical society, who have practically adopted homœopathy.

One of my patients, a mother of several children, resided the past summer with her family thirty miles distant from Albany in the country. She at first expressed unwillingness to take her family so great a distance from reliable homœopathic aid. But, on applying to the allopathic physician, a young man of decided ability residing in the neighborhood, was pleased to find that he carried a case of remedies like those used by homœopathists, and that he prescribed remedies which were quite as pleasant to the taste as any she had ever received from homœopathic sources. When twitted of being a convert to homœopathy he merely replied, "That the progress of regular medical practice had been very rapid of late years."

The trustees of the Albany (allopathic) hospital recently adopted a resolution allowing physicians of any school the privilege of treating pay patients at the hospital. The adoption of a provision so eminently just and reasonable is a result of the influence of public sentiment in behalf of *liberty of opinion and action*, as against the illiberality and sectarianism of the allopathic school, which, hitherto has been the principal barrier to the acceptance and adoption of homœopathy on the part of its more candid and unprejudiced members. Viewed in this light, it is a gratifying testimonial to the practical superiority of homœopathic principles.

The time has come, sooner than many of us anticipated, when the so-called regular school refuses longer to discipline those of its members who may adopt and practice homœopathy. This evidence of toleration on the part of that conservative fraternity is highly commendable, and worthy of imitation by our own school. While we rejoice in the promulgation of homœopathic principles, can we not easily perceive in this *coup d'etat* an element which will effectually prevent the further growth and prosperity of the homœopathic school *as a distinct and influential organization?*

Whatever the influences have been which have checked the outward development of homœopathy, it is plainly evident, that the homœopathic school, as regards the number of its openly avowed representatives, has attained its majority, and has begun to decline both in this country and in England.

The February number of the London monthly *Homœopathic Review* contains the following significant statement: "The number of those who are ready to assert their confidence in homœopathy may not have increased of late years—it may possibly have diminished—but that of those who have a confidence in homœopathy which they lack the courage to assert, has increased to an extent we have no means of calculating."

Dr. Drysdale, in the January number of the *British Journal of Homœopathy*, writes very despondingly: "Our numbers are not only not increasing in proper ratio, not even increasing at all, nay even *actually diminishing.*"

Regarding the foregoing quotation, the writer in the *Monthly Review* offers the following explanation: "To prove his case he (Dr. Drysdale) examines the homœopathic directories issued since 1853, and if their contents were any evidence at all, his conclusions would be incontestable. But such evidence as they are capable of affording is worthless in endeavoring to estimate the extent to which homœopathy is practiced in this country, or the number of those who, more or less habitually, prescribe homœopathically for their patients."

The doctor then proceeds to relate several instances which have fallen under his own personal observation, of old school physicians who have practically adopted homœopathy, while still retaining professional good standing in their own medical associations. He then continues:

"We might multiply very many fold such illustrations of the diffusion of homœopathy, of its practice by men never suspected to have adopted it. * * * * * That such should be the case is to be regretted chiefly because it shows a great weak morale to be more prevalent in the profession than is consistent with the scientific progress of our art. But it is no evidence that the practice of homœopathy has retrograded. On the contrary, it is a step in advance; it represents a period in the history of homœopathy through which it must pass, ere it meet with a general, an acknowledged acceptance throughout the profession."

Dr. Hoyne, in an article read before the Illinois Homœopathic

Medical Association, states that the number of homœopathic physicians in Illinois has scarcely increased, perhaps actually diminished during the past five years, while the population of the State, during the same period, has more than doubled.

Dr. Bruce, in his directory for 1878, furnishes the names of nine hundred and fifty homœopathic physicians residing in the State of New York. Making allowance for numerous inaccuracies, it is probable that the actual number is not far from eight hundred, a very moderate increase if any (*Investigator*, Mar. 15, 1878), perhaps an actual decrease during the past decade; while during the ten years ending July, 1875, the population of the State increased twenty-three per cent.

The increase of population in fifteen of the Northern and Eastern counties of the State of New York, during the past ten years, is sixteen per cent., while the number of homœopathic physicians residing in those counties, has not proportionately increased, probably has not increased at all.

Dr. Bruce states in his directory, edition of 1878, that he has the addresses of over 5,000 homœopathic physicians residing in the United States. Dr. Hoyne, in his directory of 1878, also states that he has the addresses of the same number of homœopathic physicians. This is no larger than the estimated number of homœopathic physicians twelve or fifteen years ago.

After a careful examination of the most recent sources of information, we are forced to the conclusion that there is, in all probability, a gradual decrease in the number of homœopathic practitioners, and, if not an actual decrease, that the ratio of increase is far below that of the population in this country.

In view of the foregoing statements, are we not justified in concluding that the period has at length arrived for homœopathists to seriously consider whether it is desirable longer to maintain separate medical organizations and institutions? For, if the powerful influences which are now in active operation continue unchecked, will not the efficiency and influence of the homœopathic school, as a distinct body of medical men, be greatly impaired, and its ultimate disintegration merely a question of time?

If it shall be deemed important, on the part of members of the homœopathic school, to maintain separate organizations, it is a matter of very great moment that measures be speedily devised and put in operation by which larger accessions to our ranks may be secured from the younger members of the allopathic school. A large proportion of the older members are zealous advocates of the allopathic system, and few of them can be induced to adopt homœopathy; but this is not true of the younger members. These constitute the large class who, at the present time, are secretly and openly practicing homœopathy while still retaining membership in allopathic associations.

Let us carefully examine this subject and endeavor to ascertain what motives induce these young physicians to unite with allopathic medical societies. Why do they prefer allopathic fellowship rather than homœopathic? Many of the younger members of the profession have a decided predilection for homœopathy; it is obvious, therefore, that the motives which induce them to unite with allopathic societies are of great influence and power. It is also evident, that membership in homœopathic medical societies is unpopular; and that, in order to turn the tide in our favor, very decided measures must be speedily put in operation. Is it not reasonable to presume that large numbers of physicians who are now practicing homœopathically under allopathic auspices, could be persuaded to unite with homœopathic societies, where they more properly belong, and where they will obtain a clearer and more thoroughly practical knowledge of homœopathy?

In view of the importance of the new and critical situation in which our school is placed, is it not desirable, in fact necessary, that concerted action be taken and that vigorous effort be made to prevent the threatened extinction of homœopathic societies and institutions? Should we not at once open the doors of our medical societies to all educated medical men, and encourage them to unite with and assist us in promoting the advancement of medical science in all its departments! Have we pursued this liberal and unsectarian policy in the past?

Nay more, are we not at the present moment exhibiting a spirit of intolerance by rescinding liberal and unsectarian declarations of faith and practice?

Have we not given medical men to understand that they were not wanted until willing to acknowledge before the public their belief in homœopathic principles? Have we not thereby actually erected a *sectarian barrier* to full professional fellowship, which ought never to have existed, and which is now proving decidedly disadvantageous to the development of our school, and is rapidly sapping its life blood? Is it wise to longer restrict membership to those physicians only who can accurately pronounce the shibboleth of doctrinal belief?

But this is not all. Have we not, as a school, followed Hahnemann into the mazes of medical transcendentalism? Have we not, and are we not now endeavoring to associate with true homœopathy that which is false, visionary and fanciful? I refer particularly to theoretical errors of the minimum dose and dynamization of medicinal and non-medicinal substances. Although these errors of theory and practice have never been accepted by many homœopathists, yet, having never been discarded by a formal declaration to that effect, have they not largely contributed to the tardy adoption of homœopathic principles?

Ought not these important questions, at this critical period in the history of our school, to be speedily and seriously considered by the whole homœopathic profession?

---

A Fair Inference.—A lady's-maid visiting with her mistress at the residence of a celebrated surgeon, then deceased, noticed the classic invitation, "salve" upon the hall floor, and in the parlor a picture of Cleopatra applying the asp to her beautiful bosom. Whereupon, with that quick but not always correct woman's intuition about which we hear so much now-a-days, she confidently, but in all innocence inquired, "Dr. —— was a physician, was he not? I felt sure he was when I saw *salve* on the entry floor, and then that poor thing in the parlor, with her broken breast and the leech in her hand. I knew he must have been a doctor."—*Archives of Clinical Surgery.*

